# Perceptions, Expectations, and Acceptability of Digital Physical Activity Programs Among Adults with Chronic Diseases in Saudi Arabia: A Qualitative Descriptive Study

**DOI:** 10.3390/healthcare14193334

**Published:** 2026-10-06

**Authors:** Jamal M. Alzahrani, Abdulaziz M. Alodhailah, Nada Alattar, Nasser Aldosari, Rayhanah R. Almutairi, Asrar S. Almutairi, Hend N. Almutairi, Rahmah I. Albahrani, Mohammed Almutairi

**Affiliations:** 1 Exercise Physiology Department, College of Sport Sciences and Physical Activity, King Saud University, Riyadh 11451, Saudi Arabia; 2 Department of Medical-Surgical Nursing, College of Nursing, King Saud University, Riyadh 11451, Saudi Arabia; 3 Department of Community and Psychiatric Mental Health Nursing, College of Nursing, King Saud University, Riyadh 11451, Saudi Arabia; 4 Research Center, King Abdullah Medical City, Ministry of Health, Makkah 24246, Saudi Arabia; 5 Department of Community and Psychiatric Mental Health Nursing, College of Nursing, Princess Nourah bint Abdulrahman University, Riyadh 11671, Saudi Arabia; 6 College of Arts and Social Sciences, The Australian National University, Canberra, ACT 2601, Australia; 7 Department of Nursing, King Saud Medical City, Riyadh 12646, Saudi Arabia

**Keywords:** digital health, mobile health, physical activity, technology acceptance, patient perception, chronic disease, qualitative research, Saudi Arabia

## Abstract

Background/Objectives: Digital programs may extend physical activity support beyond clinic visits, but qualitative evidence on how adults with chronic diseases in Saudi Arabia perceive them is limited. This study explored perceptions, expectations, and acceptability among such adults who had continued using the physical activity function of Sehhaty, the Ministry of Health’s national health application, which served as the reference point. Methods: A COREQ-reported qualitative descriptive study was conducted in chronic disease units at three tertiary centres in Riyadh between December 2025 and February 2026. Semi-structured interviews were held with a purposive sample of 14 adults (38–72 years; 8 women, 6 men) with type 2 diabetes mellitus, hypertension, cardiovascular disease, or combinations, all of whom had used the program independently for 4–16 weeks. Recruitment and analysis proceeded iteratively across two stages (18 approached). Data were analysed using reflexive thematic analysis. Results: Four themes were developed: reframed expectations of what a technology-based presence could offer; a gap between perceived usefulness and everyday usability; the dual motivational and discouraging potential of numerical feedback; and the expectation that programs fit Islamic practice, family involvement, and climate constraints. Conclusions: For these participants, acceptability depended on ease of use, natural Arabic wording, reminders and suggestions that fit prayer times, family routines, and summer heat, and feedback that recognised health circumstances. Because all participants were sustained users, the findings do not estimate acceptability in the wider population.

## 1. Introduction

### 1.1. Chronic Disease Burden and Physical Activity in Saudi Arabia

Non-communicable diseases, particularly type 2 diabetes mellitus and hypertension, constitute a substantial public health burden in Saudi Arabia. Type 2 diabetes mellitus is highly prevalent among Saudi adults, with estimates ranging from 16.4% in a recent systematic review and meta-analysis [1] to 23.7% in an earlier national survey [2]. A systematic review and meta-analysis of Saudi studies reported a pooled hypertension prevalence of 22.7% [3]. Physical inactivity is recognised internationally as a major modifiable risk factor for non-communicable diseases and premature mortality [4,5] and is of particular concern in Saudi Arabia, where extreme temperatures and other environmental and social barriers may limit opportunities for routine physical activity. Promoting and sustaining physical activity therefore represents an important public health priority.

### 1.2. Digital Physical Activity Programs as an Emerging Response

Lifestyle counselling for chronic disease, including physical activity promotion, has traditionally relied on episodic, face-to-face contact with nurses, physicians, and allied health professionals. These encounters remain clinically valuable but are limited by their intermittent nature and by the difficulty of sustaining behavioural momentum between visits [6]. Health systems, including in Saudi Arabia, have consequently begun to look toward digital physical activity programs: mobile applications, wearable trackers, and integrated platforms that offer self-monitoring, automated reminders, and real-time feedback as a way of extending support beyond the clinic [7,8].

Digital availability alone does not guarantee that a program will be used, trusted, or sustained. Attrition from application-based interventions is common, including in chronic disease and physical activity programs [9,10]. Adoption and continued use depend on technical performance, usability, personal expectations, and contextual fit [11,12], so understanding adoption requires attention to how intended users perceive and evaluate these programs as well as to whether the programs function as designed.

### 1.3. National Digital Physical Activity Initiatives and the Program Used in This Study

Several national digital initiatives now support physical activity promotion in Saudi Arabia. The Ministry of Health’s Sehhaty application, the country’s free, unified government health platform, provides medical records, appointments, and telemedicine together with healthy-lifestyle services that include step counting [11,13]. Walk 30, the Ministry’s national walking campaign, has drawn more than 1.5 million participants since its launch [14], and its first team-based league, held in March 2025, recorded nearly two billion steps from more than 44,000 participants [15]. The Ministry’s Live Well initiative adds digital health education and lifestyle campaigns [16]. The Saudi Sports for All Federation runs parallel community events such as the Riyadh Marathon, and its leadership has linked this activity to a national rise in physical activity participation from 13% in 2018 to nearly 60% in 2025 [17]. These initiatives operate at national scale but are primarily population-facing.

The digital physical activity program used in this study was the step-tracking and physical activity function of the Sehhaty application, developed by the Ministry of Health and provided free of charge. Although Sehhaty is available to the whole population, at the study sites this function was introduced to patients by chronic disease clinic staff, so participants’ experience of it was shaped by that clinical introduction. All 14 participants had used the same program. Step counts came from the native motion sensors of participants’ own smartphones and synchronised automatically, with no manual entry or separate wearable device required. As available to participants during the study period, the program comprised five functions: (1) a daily step count with a history screen showing previous days; (2) a daily step goal displayed against the recorded count; (3) weekly progress graphs and achievement notifications when goals were reached; (4) automated reminders to be active, whose timing users could change manually in the application settings but which were not linked to prayer times or to local weather; and (5) a messaging function for contacting a healthcare provider. Participants’ accounts indicate that the daily goal did not adjust automatically to recent activity levels or to periods of illness (Section 3.4). How initial step goals were set, and what the introductory clinic session covered, were not documented in the study records (Section 4.9). Provider messaging was asynchronous rather than real-time. The application offers Arabic and English interfaces; participants’ experience of the language settings is reported in Section 3.3. After a structured introduction in clinic, participants used the program independently for 4–16 weeks before interview. The program was not designed, modified, or administered by the research team and was not evaluated as a trial intervention.

Sehhaty is updated frequently, and the features described here are those available to participants during the study period. Because the Walk 30 campaign is also tracked through Sehhaty, participants’ experience of the program may have overlapped with the campaign; participation in Walk 30 was not examined in this study. Section 4.5 considers which findings are likely to be specific to this implementation and which may apply more widely.

### 1.4. Perceptions, Expectations, and Acceptability

Three related but distinct constructs organise this study: perceptions (how users interpret and make sense of a digital physical activity program in light of their prior expectations, cultural context, and experience of chronic disease); expectations (the anticipated benefits, burdens, and fit between a program and users’ health goals and daily circumstances); and acceptability (the degree to which a program is judged appropriate, tolerable, and worth continuing to use) [18,19]. These constructs are distinct from exercise adherence, which describes whether a person follows a prescribed activity recommendation. A person might remain physically active while judging a specific digital program unacceptable, or become newly active because of features such as reminders or family sharing without any change in broader motivation. Centering perception and expectation, rather than adherence or usage metrics alone, keeps the focus on users’ own accounts of the technology. In this article, ‘digital physical activity program’ refers to the general class of application-based programs, ‘the program’ refers to the physical activity function of Sehhaty that participants used, and ‘acceptability’ refers to participants’ own judgements, formed after at least four weeks of use, of whether that program was appropriate and worth continuing to use.

### 1.5. Gap in Current Evidence, Aim, and Research Questions

Qualitative evidence on how adults with chronic diseases in Saudi Arabia and the wider Gulf region perceive, anticipate, and evaluate digital physical activity programs remains limited, despite the national initiatives described above [13,14,15,16,17]. Usage metrics such as login frequency, step totals, and retention do not capture the interpretive, cultural, linguistic, and experiential considerations that influence whether people find a program useful and acceptable [11,12,20]. These considerations need to be understood before such programs are implemented at scale for this population.

This study aimed to explore perceptions, expectations, and acceptability of digital physical activity programs among adults with chronic diseases in Saudi Arabia who had used one such program for at least four weeks, with that program serving as the reference point for their accounts. Because only sustained users were included, acceptability is examined as the judgement of people who continued using a program, not as uptake or acceptability in the wider patient population. The findings were also intended to inform future research and program design in this setting. Five research questions were addressed:

1. How do adults with chronic diseases in Saudi Arabia perceive the role and purpose of a digital physical activity program in their daily disease management?
2. What expectations do participants hold regarding how such programs should function and benefit them, and how do these expectations compare with the features they actually encountered?
3. What factors strengthen or weaken the acceptability of digital physical activity programs for participants who continued using them?
4. How do cultural, religious, familial, and environmental context shape participants’ perceptions of, and expectations for, digital physical activity programs?
5. What do participants suggest would make digital physical activity programs better aligned with their needs and expectations?

## 2. Materials and Methods

### 2.1. Study Design and Methodological Orientation

This was a qualitative descriptive study. The design was chosen because qualitative evidence in this area is limited (Section 1.5), and the intent was an in-depth, contextualised account from a purposive sample rather than statistically generalisable findings. Qualitative description stays close to the language and concerns participants themselves use and is well suited to research on how health technologies are received in everyday practice [21]. Reporting followed the Consolidated Criteria for Reporting Qualitative Research (COREQ) 32-item checklist [22]; the completed checklist and interview guide are provided together as Supplementary File S1.

The study adopted a critical realist stance: participants’ perceptions and expectations are interpretively constructed through their experience with a specific program, yet also shaped by material conditions (program functionality, device reliability), cultural context, and health system structures. Data were analysed using Braun and Clarke’s reflexive thematic analysis [23,24], which treats theme development as an active, researcher-shaped process and foregrounds reflexivity throughout. The descriptive orientation of the study governs the level of interpretation offered, which stays close to participants’ accounts; the reflexive thematic analysis framework governs how those accounts were worked into themes.

### 2.2. Research Team and Reflexivity

Interviews were conducted by J.M.A. and A.M.A., both PhD-qualified male faculty members at King Saud University with five to ten years of qualitative research experience, formal training in qualitative interviewing and reflexive thematic analysis, and prior experience interviewing adults with chronic disease. Neither had a prior clinical, professional, or personal relationship with any participant. Before agreeing to participate, individuals were informed of the interviewers’ institutional affiliation and research role and told that the interviewers were not involved in their clinical care.

The team’s combined exercise-science and nursing backgrounds shaped an initial assumption that digital tools could meaningfully support chronic-disease self-management, and a corresponding attentiveness to usability, self-care, and cultural fit during interviewing and analysis. This assumption, along with other analytic decisions, was examined throughout data collection and analysis through reflexive journals and team discussions held at least fortnightly, rather than treated as neutral background.

### 2.3. Setting, Sampling, and Recruitment

The study was conducted in chronic disease units at three tertiary care centres in Riyadh, Saudi Arabia. Data collection ran from December 2025 to February 2026 and began after ethical approval was granted on 16 December 2025 (Section 2.9). Participants were recruited through clinic nursing staff and posters displayed in waiting areas. Purposive sampling was used to seek variation in age, gender, chronic disease type, and prior digital experience.

Eligibility criteria were (1) age ≥ 18 years; (2) diagnosis of type 2 diabetes mellitus, hypertension, cardiovascular disease, or a combination of these conditions; (3) disease duration ≥ 2 years; (4) access to a smartphone capable of running the digital physical activity program; (5) formal introduction to and structured instruction in the program before independent use; (6) at least 4 weeks of independent program use at the time of interview, based on self-report; (7) fluency in Arabic or English; and (8) capacity to provide informed consent and participate in an extended interview. Exclusion criteria were acute illness at the time of interview, cognitive impairment precluding informed consent, and concurrent participation in a formal clinical trial of a physical activity intervention.

**Figure 1:**
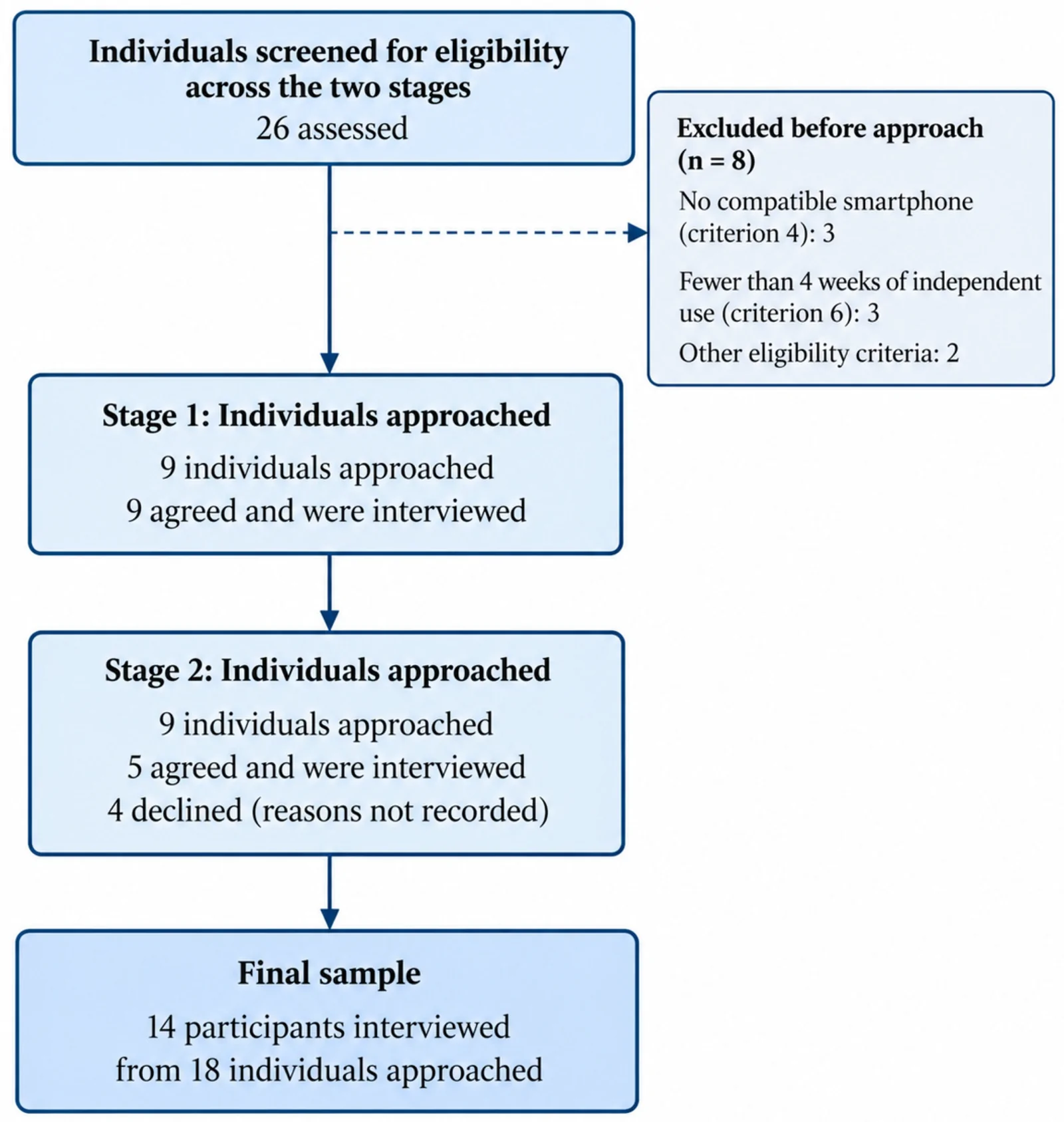
Participant recruitment flow across the two-stage purposive sampling process.

Recruitment proceeded in two stages. During the initial stage, nine individuals were approached, and all agreed to participate. Following an initial review of these interviews, the research team identified areas in which additional perspectives could strengthen the depth and variation of the dataset. During the second stage, nine further individuals were approached; five agreed to participate and four declined. Reasons for declining were not recorded. The additional interviews followed the same eligibility criteria, semi-structured approach, and data collection procedures as the initial interviews. Of the 26 individuals screened for eligibility across the two stages, 8 were excluded before approach: 3 did not meet criterion (4) (no compatible smartphone), 3 did not meet criterion (6) (fewer than 4 weeks of independent use), and 2 did not meet other eligibility criteria. Fourteen participants were therefore interviewed in total from the 18 individuals approached. Figure 1 summarises this recruitment flow.

The sample size was not fixed in advance using formal data-saturation criteria. Consistent with reflexive thematic analysis, dataset adequacy was judged against the information needed to address the research questions rather than a predetermined numerical threshold. Data collection and analysis proceeded iteratively, with insights from earlier interviews informing subsequent data collection. The second-stage interviews allowed consistency and variation in participants’ experiences to be examined against the developing themes.

Recruiting participants who were already using a digital physical activity program was a deliberate methodological choice. It enabled participants to draw on direct, situated experience rather than hypothetical impressions, while the study aim and analysis remained focused on the broader question of how such programs are perceived and might be adopted. The same choice defines the scope of the acceptability findings: they describe how people who adopted and continued using a program judged it, and cannot explain why others declined, could not start, or stopped. The consequences of this choice for interpretation are addressed in Section 4.7 and Section 4.9.

### 2.4. Participants

Fourteen adults took part (Table 1): eight women and six men, aged 38–72 years (mean 55.6, SD 11.1). Nine participants had type 2 diabetes mellitus, eight had hypertension, and three had cardiovascular disease; five had two or more concurrent conditions. Disease duration ranged from 2 to 18 years (mean 9.0, SD 5.0). Prior experience with digital technology ranged from minimal use, such as basic phone calls and messaging, to daily use of multiple applications. Duration of program use at the time of interview, as self-reported on the pre-interview questionnaire, ranged from 4 to 16 weeks (mean 10.0, SD 3.7). Eleven participants were Saudi nationals, and three were expatriate residents with long-term residency status. Among the three participants with cardiovascular disease, the specific diagnoses were coronary artery disease, heart failure, and a previous myocardial infarction.

### 2.5. Data Collection

Semi-structured, one-to-one interviews were conducted by J.M.A. and A.M.A. (Section 2.2), either in person at the study sites or online by videoconference, according to participant preference. Only the participant and the interviewer were present; no other individuals attended. Interviews were conducted in Arabic and were audio-recorded with participants’ permission. All 14 participants, including the three expatriate residents, chose to be interviewed in Arabic. Each participant was interviewed once; no repeat interviews were conducted. Demographic and clinical information was collected using a brief questionnaire administered before each interview. The interviewers recorded field notes during and immediately after each interview on contextual and non-verbal observations and initial analytic impressions.

The interview guide was developed iteratively by the research team, informed by a review of the literature on mobile health acceptability, and input from a small advisory panel comprising clinic-based nurses and two patient representatives with chronic disease experience. The guide was piloted before formal data collection to assess the clarity, sequencing, and flow of the questions, and minor wording refinements followed. The final guide comprised 20 open-ended questions and associated probes across five topic areas: (1) initial perceptions and expectations on learning about the program; (2) experiences with application navigation, interface design, and usability; (3) perceptions of activity tracking and feedback mechanisms; (4) integration of the program into daily routines, including cultural, religious, familial, and environmental contexts; and (5) expectations regarding healthcare provider involvement, perceived benefits, overall acceptability, and suggested improvements. These topic areas guided the interviews and did not constitute predetermined analytic themes. The complete guide is provided in Supplementary File S1.

Interviews lasted 45–75 min.

### 2.6. Language and Translation

All interviews were transcribed verbatim in Arabic before translation into English. Translation was undertaken by a bilingual member of the research team, with attention to conceptual and cultural equivalence rather than literal, word-for-word rendering, consistent with recommended practice in cross-language qualitative research [25]. A second bilingual team member independently checked each translated transcript against the Arabic original for accuracy and fidelity of meaning. Discrepancies were resolved through discussion and, where necessary, by returning to the original audio recording. Terms without direct English equivalents, including references to Islamic prayer times such as Asr and Maghrib, were retained in their original Arabic form within participant quotations. Independent back-translation into Arabic was not undertaken; this is acknowledged as a limitation in Section 4.9.

### 2.7. Data Analysis

Audio recordings were transcribed verbatim within one week of each interview. Transcripts were independently reviewed by two analysts, who cross-checked them against the corresponding audio recordings to verify accuracy and completeness; discrepancies were resolved with reference to the original recording.

Braun and Clarke’s six-phase reflexive thematic analysis was then applied [23,24]. Analysis was iterative, with the analysts moving between transcripts, codes, candidate categories, and developing themes throughout.

Phase 1: Familiarisation. The analysts immersed themselves in the data through repeated reading of the transcripts and memo-writing, documenting initial impressions, recurring ideas, areas of variation, and potentially meaningful patterns.

Phase 2: Generating initial codes. Each of the two analysts (J.M.A. and A.M.A.) independently coded all 14 transcripts, generating separate sets of initial descriptive codes to capture discrete segments of meaning relevant to participants’ perceptions, expectations, experiences, and acceptability. Coding was primarily inductive, with codes generated from participants’ accounts rather than applied through a predetermined framework. The analysts then compared their codes, treating differences as alternative readings rather than disagreements to be resolved (final paragraph of this section), while recognising that the data had been generated through a guide organised around predefined topic areas.

Phase 3: Constructing candidate themes. Initial codes were compared and organised into broader candidate categories representing shared patterns of meaning. Regular analytic meetings between the two analysts provided reflexive spaces to examine interpretive assumptions, consider alternative readings, and refine the scope and organising logic of the candidate categories. These discussions were not intended to establish inter-rater reliability.

Phase 4: Reviewing candidate themes. Candidate themes were reviewed against the associated coded extracts and then against the full dataset, assessing internal coherence, distinctiveness, and explanatory contribution. Themes were refined iteratively, with some categories merged where substantial conceptual overlap was identified, and others differentiated where further variation was identified.

Phase 5: Defining and naming themes. The analysts defined the scope, central organising concept, and narrative essence of each final theme. Theme names were developed to reflect the underlying meaning of participants’ accounts while recognising that theme construction involved interpretation by the research team. Four themes were produced.

**Figure 2:**
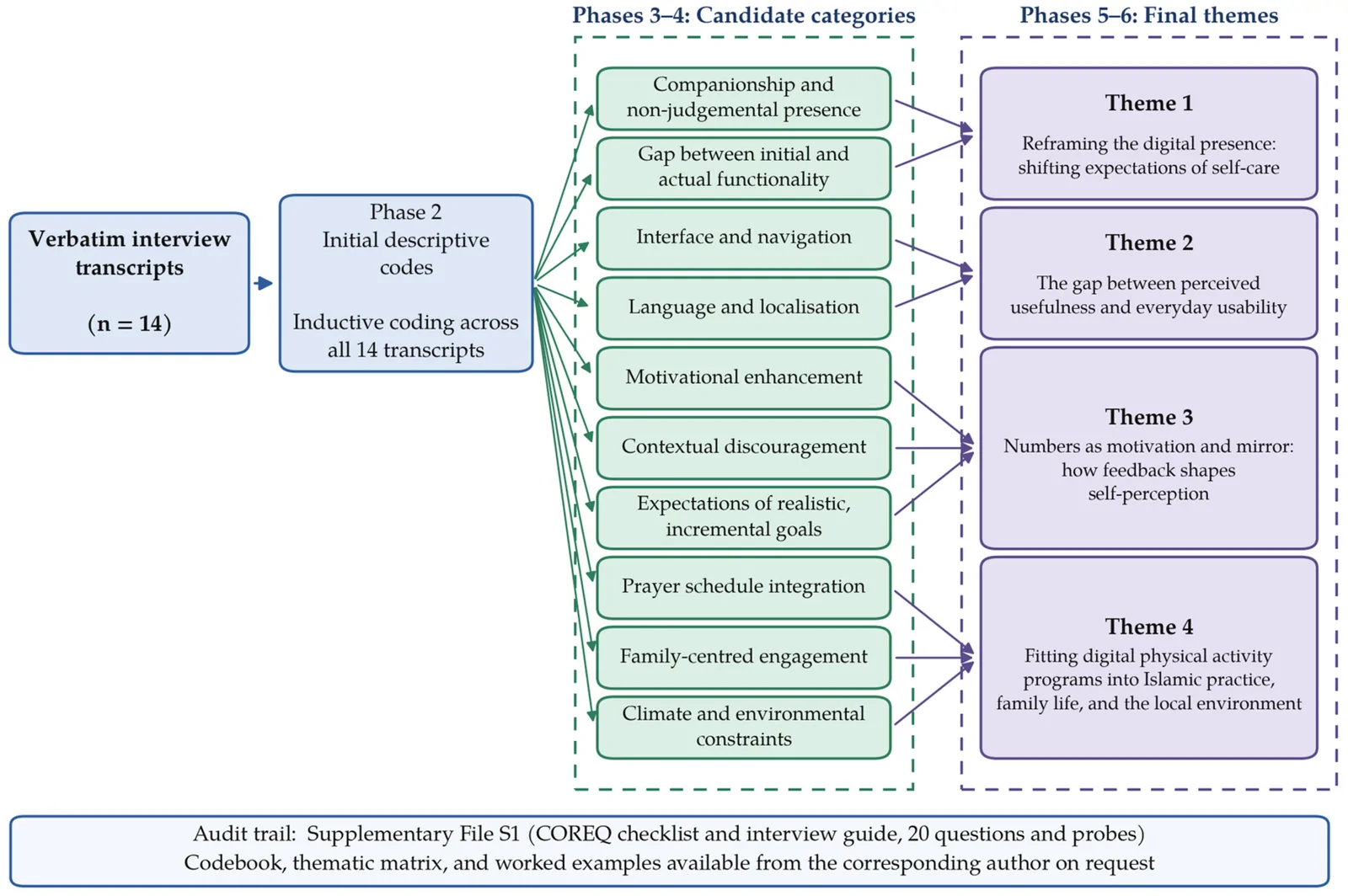
Overview of the reflexive thematic analysis process, showing the analytic progression from verbatim interview transcripts and initial descriptive coding through candidate categories and iterative theme development to the four final themes reported in Section 3.

Phase 6: Producing the report. The final themes were synthesised into a coherent narrative and used to structure Section 3 and Section 4. Illustrative quotations were selected to demonstrate the depth, range, and variation of participants’ experiences while maintaining confidentiality. Figure 2 summarises the progression from transcripts through initial codes and candidate categories to the four final themes; the codebook, thematic matrix, and worked examples linking raw-data extracts to codes, categories, and themes are available from the corresponding author on reasonable request.

Given the modest dataset and the study’s emphasis on close engagement with participants’ accounts, data were managed manually using Microsoft Word and Microsoft Excel rather than dedicated qualitative data-analysis software. The software served as an organisational aid for transcripts, coding decisions, analytic memos, and developing themes; all analytic judgements were made by the analysts.

Reflexive journals and the fortnightly team meetings (Section 2.2) were used throughout to record analytic decisions, examine positionality, and test emerging interpretations against the full dataset.

Analysis of all 14 transcripts produced 10 candidate categories, which were reviewed and refined into the four final themes reported in Section 3.

From individual coding to the final thematic structure. The two analysts’ independent code sets were compared at the first analytic meetings. Codes that described the same segment of meaning in different words were combined under a shared label. Codes that reflected different readings of the same extract were kept side by side, and each analyst recorded in a memo the reasoning behind their reading. Each pair of readings was then tested against the rest of the dataset: the analysts returned to all extracts relevant to it and asked which organising concept accounted for the range of participants’ accounts, including those that did not fit. Where one reading was better supported, it became the organising concept for the candidate category; where both were supported, the tension was kept visible in the report rather than resolved. Extracts describing family help with the application illustrate the second outcome. They could be read as evidence that the program was not usable without help, or as a culturally expected form of shared health management. Both readings were supported, so family involvement is reported under Theme 2 (usability and independence), under Theme 4 (family-centred engagement), and as a point of divergence in Section 3.6. These decisions were recorded in the audit trail. The final structure of ten candidate categories and four themes was agreed by both analysts and discussed with the wider research team before the report was written.

### 2.8. Rigour and Trustworthiness

Trustworthiness was addressed using the criteria of Lincoln and Guba [26].

*Credibility* was supported through in-depth individual interviews that allowed each topic to be explored at length, peer debriefing (regular discussion among the research team and with the two patient advisors), and member checking in which preliminary themes and illustrative quotations were shared with four participants who had agreed to be re-contacted, for feedback on accuracy and resonance. Full transcripts were not returned to participants for comment or correction, and the member-checking feedback was treated as an additional interpretive perspective rather than as verification of the analysis.

*Dependability* was supported through an audit trail documenting analytic decisions, transparent reporting of coding decisions and theme construction, and reflexive engagement by two analysts whose differing readings were documented rather than reconciled; agreement was sought only on the final thematic structure (Section 2.7).

*Transferability* was supported through detailed description of setting, participants, and context, allowing readers to judge applicability to other populations and settings; Section 4.7 sets out the settings and populations to which the findings are more and less likely to transfer.

*Confirmability* was supported through the reflexive journals, explicit attention to potential researcher influence, and documentation of the frameworks informing the study.

### 2.9. Ethical Approval and Informed Consent

Ethical approval was obtained from the King Saud University Institutional Review Board (Reference KSU-HE-25-1489, approved 16 December 2025) before any data were collected; this approval covered all three study sites. The study was conducted in accordance with the Declaration of Helsinki. Written informed consent was obtained from all participants before the interview. Participants were informed that participation was entirely voluntary, that they could withdraw or decline to answer specific questions without consequence, that interviews would be audio-recorded with their permission, that anonymised quotations might be published, and that all data would be kept confidential and identified only by coded participant identifiers. No incentives were offered beyond light refreshments during interviews.

## 3. Results

### 3.1. Overview

Analysis of the 14 interviews produced four themes, each comprising two or three subthemes, that together characterise how participants perceived and formed expectations of digital physical activity support. Theme 1 addresses research question 1; Theme 2 addresses research questions 2 and 3; Theme 3 addresses research questions 2, 3, and 5; and Theme 4 addresses research questions 4 and 5. Participants’ suggestions for improvement (research question 5) are reported within Themes 2, 3, and 4 rather than as a separate theme, because they arose in participants’ accounts as responses to specific difficulties rather than as free-standing recommendations. Table 2 summarises the thematic structure and the participants whose extracts are presented for each subtheme. Extracts from all 14 participants appear below. Throughout this section, statements about acceptability refer to the judgements of these 14 participants, all of whom had continued using the program for at least four weeks. Participants said little about healthcare provider involvement despite a direct question on this topic, and these accounts were not developed into a theme.

### 3.2. Theme 1: Reframing the Digital Presence: Shifting Expectations of Self-Care

Companionship and non-judgemental presence. Several participants described the program not as an impersonal tool or mechanical counter but as a quasi-social presence that altered their expectations of what a digital program could offer and of who, or what, could provide supportive care. Some expressed surprise that a program could provide continuous encouragement outside the clinic without conveying judgement. Participant 3 described the feedback in these terms:

“I thought it would just count steps, like a pedometer. But the reminders, they’re gentle, not bossy. It just says, ‘today you did 5000 steps,’ and if tomorrow I do 6000, it notices. I didn’t really expect that from a machine.”

Participant 7 described a sense of support between appointments:

“My nurse always tells me I must exercise, and I believe her, but between appointments I forget, or I make excuses. This program, it’s a bit like having someone who checks on me. Not angry, not lecturing. It asks about my day through the numbers, if that makes sense. At first I thought, ‘it’s just an app,’ but after a while it felt like having someone who cares.”

Participant 10 described a gradual shift in how the program became part of everyday life:

“At first I didn’t think much about it. I thought, okay, it counts the walking, that’s it. But after some time I started looking at it myself. Sometimes I don’t even remember why I opened it, I just check. It’s become part of my day, I guess.”

Participant 11 described a low-pressure form of encouragement:

“I liked that it was there every day. Even if I didn’t do much, I could see what happened. It doesn’t tell me off. Sometimes that’s enough, just to see it and think, okay, tomorrow I’ll try again.”

Gap between initial and actual functionality. Alongside these accounts, some participants had initially expected the program to be more “intelligent” or capable of personalised advice, and described mild disappointment when it could not adapt recommendations to their specific health circumstances, such as during periods of illness, without provider input. Participant 5 described this expectation:

“At first I thought it would know more about my condition, you know? Like if I wasn’t well that day, maybe it would change what it tells me to do. But it doesn’t really know that. It just gives you the same thing.”

Participant 9’s account, presented under Theme 3 (Section 3.4), makes the same point in relation to illness: during a period of cardiac symptoms, the participant wanted the program to recognise that low step counts could have a medical reason rather than reflecting a lack of effort.

### 3.3. Theme 2: The Gap Between Perceived Usefulness and Everyday Usability

Despite positive perceptions of the program’s purpose, participants described friction between its perceived usefulness and their everyday experience of using it.

Interface and navigation. Participant 1 described the first week of use:

“The first week was very confusing. The buttons, I didn’t know what each one did. I kept opening screens by accident. My son had to sit with me: ‘this button is for steps, this one’s history, this one’s reminders.’ Once I learned it, I could use it fine. But it shouldn’t need a young person to sit and teach you, you know? Some of the words are only in English too, and my English isn’t strong.”

Participant 12 described a similar difficulty and a related dependence on family support:

“Some things were easy, but then suddenly I didn’t know where to go. I would press something and another page comes up. I don’t want to keep asking my daughter every time. If I’m using it, I want to know I can do it myself.”

Participant 13 focused on the density of information on screen:

“I don’t need many things on the screen. Just show me what I need. Steps, maybe the goal, and what I did yesterday. When there are too many things, I don’t know what I’m supposed to look at.”

Language and localisation. Several participants said that although the program supported Arabic, some screens appeared in English, particularly when the Arabic interface had not been selected, and some Arabic wording read awkwardly. Participant 8’s comments concerned the voice and tone of the Arabic wording and these English screens, which Participant 8 linked directly to trust:

“The main screens are in Arabic, which is good. But sometimes there’s a button in English, or the Arabic sounds strange, you know, not how people actually talk. I want a program for Saudi people to sound Saudi, not just translated. Honestly, it makes me trust it less when the wording feels unnatural like that.”

Participants 1 and 12 linked these difficulties to whether they could use the program without help, and both described reliance on family members for technical support as a threat to their independence. Other participants viewed family involvement positively (Section 3.5).

### 3.4. Theme 3: Numbers as Motivation and Mirror: How Feedback Shapes Self-Perception

Participants described visual feedback components, including step counts and weekly progress graphs, as particularly influential in shaping perceptions of their own capability and effort. The direction of that influence, motivational or discouraging, was highly individual and context-dependent.

Motivational enhancement. Participant 2 described:

“I can see my steps every day. Most weeks I’m improving, the graph goes up. Last week was around 35,000 total, my best so far, up from maybe 28,000 the week before. That made me happy. I showed my husband and daughter, and they said, ‘Mama, you’re doing so well!’ It’s silly maybe, but that graph made me proud of myself. Now I check it most evenings before I sleep, and if I’m under my goal I try to walk a bit more.”

Participant 10 described a more qualified response, in which the number confirmed what was already known rather than adding motivation:

“Sometimes I see the number and think, oh, that’s not bad. Other days I look and say, that’s really low. It depends on the day. If I’m tired or my knee is hurting, I already know why it’s low. Seeing the number doesn’t change that.”

Contextual discouragement. Participant 9 described the same kind of display during a period of cardiac symptoms:

“When I had my heart symptoms last month, I couldn’t walk far. My steps dropped to maybe 4000 a day. The app kept showing these low numbers every morning, like it was reminding me I’m sick. I know I couldn’t exercise, my doctor said rest. But seeing it like that, low number after low number, it made me feel worse about myself. I stopped checking the app that week, actually. It should understand, sometimes there’s a medical reason, it’s not just laziness.”

Expectations of realistic, incremental goals. Participant 14 linked acceptability of feedback to the size of the increment demanded:

“If it says you have to reach this big number every day, sometimes I just leave it. Maybe give me something small first. If yesterday I did 3000, don’t tell me I need 10,000. Tell me, okay, try 3500. That feels possible.”

Numerical feedback therefore operated both as motivational reinforcement and as a potential source of inadequacy, depending on whether participants attributed low activity to personal effort or to circumstantial or health-related constraints. Participants expected a digital physical activity program to contextualise feedback, or to offer alternative views highlighting recovery and sustainable incremental progress rather than absolute performance.

### 3.5. Theme 4: Fitting Digital Physical Activity Programs into Islamic Practice, Family Life, and the Local Environment

A distinctive theme concerned how participants expected the program to align with, rather than compete with, aspects of Saudi daily life: Islamic religious practice, family structures, and climate-related constraints.

Prayer schedule integration. Participant 4 described the mismatch between reminder timing and the structure of the day:

“The app sends reminders at random times, one day 2 p.m., the next 3 p.m., I don’t know why. I’d prefer if I could set them around prayer times, after Asr, before Maghrib, when it’s cooler, I could go walk then. But I have to change the settings myself. I don’t think the designers really thought about how our day is structured, you know. We don’t just look at the clock, we look at prayer times.”

Participant 11 made a similar point about when activity is actually feasible:

“If you tell me to walk at two in the afternoon, no, I’m not going outside at that time. After prayer, maybe, or in the evening. That’s when people are actually free. The reminder should understand our day.”

Several participants described manually reconfiguring reminder schedules to align with prayer times and post-prayer periods, when physical activity was more culturally acceptable and environmentally feasible, particularly in hot weather. This required technical proficiency and initiative beyond what some participants possessed, creating barriers for less digitally experienced users.

Family-centred engagement. Contrary to assumptions embedded in individualistic application design, several participants expected and preferred that family members be aware of and involved in their activity monitoring. Participant 6 described this as an extension of ordinary family conversation:

“My daughter helped me download the app and set it up. Now she asks me sometimes, ‘Papa, how many steps today?’ It’s become part of how we talk. I like that, in our culture family gets involved in health things. Actually, I started checking my goals more carefully once she was involved. Maybe the program could let her see my progress too, or get reminders, so she’s more part of it.”

Participant 12, however, described the tension between appreciating family assistance and wanting to remain independent:

“I don’t mind my daughter helping me. But I don’t want to call her every time something changes. Some things I should be able to do myself.”

Family involvement could therefore function both as a source of motivation and practical support and as an indication that the program was not sufficiently accessible for independent use.

Climate and environmental constraints. Participant 7 described the limits imposed by summer temperatures and the need for suggestions suited to local conditions:

“In summer it’s like 48, 50 degrees outside, honestly. I can’t walk outside, I stay in with the AC. I’d want the program to suit our conditions here and suggest something indoors when it’s that hot, or at least say something about it.”

Participant 13 emphasised the need for realistic indoor alternatives:

“In the summer, walking outside is difficult. If the program just says walk outside, I don’t follow it. Give me something I can do at home. Even ten minutes, something simple.”

Participants therefore viewed the program as needing to account for the environmental conditions in which physical activity actually occurs. Rather than simply recommending activity, they expected suggestions to reflect local climate and the practical options available to them.

### 3.6. Variation and Divergent Cases

Participants’ accounts were not uniform. The same program feature could support one participant and discourage another, depending on health status, expectations, digital confidence, family circumstances, and daily routine. The extracts presented in Section 3.2 to Section 3.5 illustrate this variation without requiring further data.

The clearest divergence concerned numerical feedback. Participant 2 described a rising weekly graph as a source of pride that prompted further walking, while Participant 9, during a period of cardiac symptoms, described the same kind of display as a daily reminder of illness and stopped opening the program for a week. Participant 10 occupied an intermediate position, treating the number as information already known rather than as either encouragement or reproach. Participant 9’s account functions as a disconfirming case: feedback designed to encourage activity instead produced discouragement and temporary disengagement. It does not overturn the broader pattern that participants valued feedback, but it locates the effect of feedback in the participant’s health circumstances rather than in the feature itself.

Participants also differed in how independently they could use the program. Participant 1 and Participant 12 both required family assistance but framed it differently: Participant 1 described her son’s instruction as a workable, if undesirable, route to competence, whereas Participant 12 experienced repeated recourse to a daughter as an erosion of independence. Participant 6, by contrast, described a daughter’s involvement as a welcome extension of family life into health management and wanted the program to support it directly. Family involvement therefore appeared both as a practical requirement created by usability problems and as a valued source of encouragement.

Expectations of cultural adaptation likewise operated at different levels. Participant 8 was concerned with Arabic that read as translated rather than spoken, while Participants 4, 7, 11, and 13 described requirements reaching further into the organisation of the day: reminder timing anchored to prayer rather than to the clock, and activity suggestions that acknowledged summer temperatures. Cultural fit was therefore not a single requirement but a graded set of expectations extending from terminology through to the structure of daily life.

Each quotation presented above is drawn from a distinct passage of a transcript; duplicate or reworded versions of the same extract were not treated as separate quotations.

## 4. Discussion

### 4.1. Perceived Presence and Reframed Expectations of Self-Care Support

Several participants (Participants 3, 7, 10, and 11) came to describe the program in social terms, as something that checked on them and noticed their effort, although Participants 3 and 10 had initially expected little more than a step counter. This view developed through use rather than being an expectation participants brought to the program, and it was not described by every participant. This is consistent with evidence that anthropomorphic cues and perceived social presence shape trust in, and acceptance of, interactive health technologies [27].

For these participants, the shift from anticipating an impersonal counter to experiencing an encouraging presence appeared to make self-monitoring feel less burdensome and more routine (Participant 10). The program also incorporated behaviour-change components such as self-monitoring, feedback, reminders, and goal setting, which are recognised behaviour change techniques [28]. The converse pattern, in which gaps between expected and actual functionality (for example, inability to adapt advice during illness; Participant 5) produced disappointment, suggests that clear communication about a program’s capabilities and limitations at the outset would help calibrate expectations.

### 4.2. Usability, Language, and Implementation Considerations

For the participants who encountered them, interface confusion and language inconsistencies were more than minor inconveniences: Participant 8 said that awkward or untranslated Arabic reduced trust in the program, and Participants 1 and 12 described needing family help to use it. The accounts reported here concern usability and language rather than technical failures such as crashes or lost data, so they do not speak to technical reliability. They are consistent with evidence that usability problems are a common reason for disengaging from health applications and a recognised barrier to their adoption [29,30].

The need for family members to mediate technology use, described by Participants 1 and 12, raises a further implementation consideration. Family involvement can enhance motivation, but reliance on it may undermine autonomy and limit reproducibility across populations with varying family support. Older adults are known to meet specific usability difficulties with health technologies [31], so health systems considering digital physical activity programs at scale may wish to weigh whether structured digital literacy support, such as brief in-person training for older or less experienced users, could reduce early disengagement and support independent use.

Prior digital experience is likely to have shaped these perceptions. The sample ranged from four participants whose phone use was limited to calls and text messages to four who used multiple applications daily (Table 1). Difficulty finding features, dependence on family for setup, and the effort of manually reconfiguring reminders around prayer times (Section 3.5) are barriers that weigh most on less experienced users, whereas more experienced users are better placed to work around them. The analysis was not structured to compare accounts by level of digital experience, and 14 accounts could not support such a comparison. The eligibility criteria also matter here: even participants with minimal experience had managed at least four weeks of use, so people whose limited digital experience prevented them from starting or continuing are not represented. The usability barriers reported here may therefore understate those faced by less experienced patients in routine care [11,31].

The language findings warrant particular attention. Arabic main screens were appreciated, but English labels and wording that read as translated undermined credibility. Evaluations of Arabic health materials show that vocabulary and sentence structure affect how easily readers understand them [32], so wording that reads naturally to Saudi users, rather than generic translation, is likely to matter for whether users judge a program acceptable; among these sustained users, wording that read as translated was already enough to reduce trust.

### 4.3. Feedback Design and Its Psychological Weight

These findings identify a tension in digital health feedback design: the same numerical mechanism that enhanced motivation in some participants produced a sense of inadequacy in others, largely depending on whether low activity was attributed to personal capacity or to circumstantial constraints such as illness or climate. This reflects the wider literature on the double-edged nature of performance feedback, which can trigger avoidance rather than persistence when it is presented without context [33].

Feedback that acknowledges constraints while still reinforcing incremental progress, or that offers users choice about what data are visualised, may make feedback acceptable to a wider range of users, although this study shows only how sustained users responded to the feedback they received. Participants did not simply want higher numbers; they wanted feedback recognising effort relative to circumstances. Given the established health benefits of physical activity [34], these findings support designing feedback that helps people pursue activity goals without disregarding temporary clinical or environmental constraints.

### 4.4. Cultural and Contextual Fit as a Precondition for Adoption

Participants’ accounts show how cultural context shaped their judgements of acceptability in ways that designers may not anticipate even in a nationally developed application, a point Participant 4 made directly. Prayer schedule integration, family involvement in monitoring, and explicit acknowledgment of climate constraints were not trivial personalisations for these participants but expectations rooted in how daily life is structured, consistent with evidence that health interventions tailored to Islamic practice are associated with better outcomes [35].

That participants themselves undertook adaptation, manually reconfiguring reminders around prayer times, demonstrates resourcefulness but also indicates that failure to build cultural adaptation into core design generates user-led workarounds that will not be feasible for everyone. For programs used at national scale, treating cultural context as a design input rather than a localisation afterthought could improve acceptability [36], although whether it does so beyond the sustained users interviewed here would need testing.

The expectation that family members be visible within the digital ecosystem also reflects the importance of family participation in health-related decisions in Saudi settings [37]. Standard mobile health tools may assume an individual user; family-aware features such as privacy-respecting sharing, family goal-setting options, or asynchronous family communication may therefore improve acceptability when, and only when, the patient wishes family members to be involved. Whether this extends to the broader Middle East is beyond what these data can establish.

### 4.5. Variation Between Participants, Clinical Heterogeneity, and Program-Specific Findings

The variation described in Section 3.6 has direct design implications. Whether step counts and progress graphs encouraged or discouraged a participant depended on the attribution made for a low figure (Participants 2 and 9), and family involvement both supported and constrained independent use. Perceived usefulness was therefore conditional: the same feature supported engagement for one participant while producing frustration, discouragement, or dependence for another. Digital physical activity programs for this population need to accommodate differences in health circumstances, digital confidence, family involvement, and daily routines rather than assume a single user profile.

The clinical heterogeneity of the sample bears on this conditionality. Participants had type 2 diabetes mellitus, hypertension, cardiovascular disease, or combinations of these (Table 1), conditions that differ in how physical activity is prescribed and in how safely a person can increase it. For people with coronary artery disease, heart failure, or a previous myocardial infarction, a fall in step count may reflect a clinical instruction to rest, as Participant 9’s account illustrates, and step goals may need to be set within limits agreed with a clinician or a cardiac rehabilitation team. For people with diabetes, activity advice is tied to glycaemic management and to precautions against hypoglycaemia, and for people with several conditions the constraints of each may combine; Participant 10 also described musculoskeletal pain limiting activity regardless of diagnosis. Goal and feedback logic designed for a single generic user is therefore poorly matched to this population. The analysis was not organised by diagnosis, and the sample is too small to compare accounts across conditions; how perceptions differ between these clinical groups requires studies designed for that comparison.

Some findings are tied to this particular program, while others are likely to apply to digital physical activity programs more widely. English screens and labels when the Arabic interface was not selected, reminder timing unrelated to prayer times, reminders that did not take summer heat into account, and a goal display that did not respond to illness are features of the program participants used, and a program built differently might not attract the same complaints. Because the physical activity function sits within Sehhaty, a platform that also handles appointments, medical records, and sick leave, some complaints about navigation and crowded screens (Participants 12 and 13) may partly concern the application as a whole rather than its physical activity function. The expectations beneath those complaints are less program-specific: that the interface can be used without help, that Arabic reads naturally, that feedback recognises health circumstances, and that reminders and suggestions fit the structure of the day and the climate. These expectations are the more portable findings; the specific complaints show how one implementation fell short of them. All of these are patterns across 14 accounts from participants who had remained engaged with the program, and they would require testing in a larger and more varied sample that includes people who discontinued use.

### 4.6. Implications for Design and Implementation

The themes point to specific design and implementation measures for digital physical activity programs for people with chronic diseases in Saudi Arabia. Table 3 links each theme to these measures, and four considerations are discussed below.

First, integration with clinical care. The findings reported here do not address participants’ use of the provider messaging function, so they offer limited direct evidence on clinical integration. Two points nonetheless arise from participants’ accounts. Some participants expected the program to adapt advice during illness and were disappointed that it could not do so without provider input (Section 3.2), which suggests that the introductory session in clinic should state plainly what a program will and will not do. Participant 9’s account also indicates that clinicians who advise rest should be able to pause or lower goals, so that the program does not contradict clinical advice. Whether clinician introduction or endorsement influences uptake or continued use was not tested in this study.

Second, usability and language. Programs should keep the main screen to a few core items, such as steps, the daily goal, and the previous day’s total, as Participant 13 requested. They should be fully available in Arabic, with the Arabic interface set as the default and wording reviewed by Saudi users rather than translated directly, since awkward or partly English wording reduced trust (Participant 8) and made the program harder to use for participants with limited English (Participant 1). User testing with patients who have minimal digital experience, together with a brief supported setup session for those who need it, could reduce dependence on family members for basic operation. Reliable functioning across commonly used devices should also be confirmed before wider implementation, although reliability was not a focus of participants’ accounts.

Third, cultural, religious, and environmental adaptation built into core design. Concrete options include reminder presets anchored to locally calculated prayer times (for example, after Asr or before Maghrib), which would remove the need for users to reconfigure settings themselves (Participants 4 and 11); heat-aware suggestions that switch to short indoor activities when outdoor temperatures are high (Participants 7 and 13); and optional, patient-controlled sharing of progress with a named family member, offered only when the patient wants family involved (Participant 6). Adjusting reminders during Ramadan, when daily routines change, would follow the same principle, although participants did not raise it. Developing these features with patients, healthcare professionals, and community stakeholders [36] would reduce reliance on user-led workarounds that less experienced users cannot manage.

Fourth, feedback and goal setting. Goals could start from a user’s recent baseline and rise in small increments, as Participant 14 proposed, and users or clinicians could mark periods of illness so that feedback emphasises recovery rather than shortfall (Participant 9). Offering a choice between daily totals and longer-term trends would give users who find daily numbers discouraging another way to see progress. None of these measures was tested in this study; they are design hypotheses drawn from the accounts of sustained users and should be evaluated with a broader population, including people who stop using such programs.

### 4.7. Transferability of the Findings

The findings come from 14 adults attending chronic disease units at three tertiary centres in Riyadh, all of whom had been introduced to the program in clinic and had continued to use it for at least four weeks. Transferability should be judged against these conditions. The findings are most likely to apply to urban tertiary care settings in Saudi Arabia and neighbouring Gulf states where clinical staff introduce digital physical activity programs to adults with type 2 diabetes mellitus, hypertension, or cardiovascular disease. Expectations concerning prayer-time scheduling and family involvement are likely to be relevant in other Muslim-majority and family-oriented settings, and the climate findings in other hot regions, although the form these expectations take will vary. Transfer is less certain to primary care and rural settings, where access to devices, digital literacy, and clinic support may differ; to Saudi regions with different climates and daily routines; to people who use Sehhaty’s activity features on their own or through national campaigns such as Walk 30, without clinical introduction; and to people who declined or stopped using a program, whose views are absent. The description of participants (Table 1), the program (Section 1.3), and the interview guide (Supplementary File S1) is intended to let readers make these judgements for their own settings.

### 4.8. Strengths

This study provides an in-depth qualitative account of perceptions and expectations grounded in participants’ direct experience of a digital physical activity program in Saudi healthcare settings. The interviews elicited interpretive, cultural, linguistic, and usability considerations that usage metrics alone would not capture. Reflexive thematic analysis [23,24], an audit trail, reflexive journaling, peer debriefing, and participant feedback on preliminary themes supported transparency and credibility. Variation in age, chronic disease profile, duration of program exposure, and prior digital experience broadened the range of perspectives represented within a small purposive sample.

### 4.9. Limitations

The sample of 14 participants from three tertiary centres in Riyadh is geographically concentrated and was assembled for depth rather than generalisability; the implications for transferability are set out in Section 4.7.

The eligibility criteria carry a substantive selection bias. All participants had used the program for at least four weeks, so the design excludes people who declined to begin using it, who could not install or operate it, who lacked a compatible smartphone, or who discontinued early for technical, clinical, linguistic, cultural, or motivational reasons. Those are the accounts most directly relevant to non-acceptance and to failed implementation. The finding that the program was generally regarded as acceptable should therefore be read as an account from sustained users, not as an estimate of acceptability among all patients offered the program.

The relatively short window of use (4–16 weeks) does not capture expectations and acceptability over longer-term use. As a single-time-point interview study, it cannot track how perceptions evolve or determine which design features most affect acceptability; the themes reported here are better treated as hypotheses for subsequent testing than as confirmed findings. Because participants’ accounts were formed through experience of a single program, the findings reflect perceptions of one implementation and may not extend uniformly to all digital physical activity programs. Sehhaty is updated frequently, so the features described in Section 1.3 reflect the version available during the study period and may differ from later versions. Duration of use was self-reported and may be imprecise, particularly because the application records steps passively once installed. The program was also one function of a multi-service national application that many people use for other purposes; participants’ prior use of Sehhaty for other services is not reported, so how that familiarity shaped their initial expectations and usability judgements cannot be assessed. The description is also incomplete: the study records did not document how initial step goals were set or what the introductory clinic session covered, so the influence of goal setting and initial instruction on participants’ experiences cannot be assessed.

Translation accuracy was checked by a second bilingual team member rather than by independent back-translation. Although discrepancies were resolved against the original audio, some shift or loss of nuance between Arabic and English cannot be ruled out.

Both interviewers were men, and eight of the 14 participants were women. Although participants chose between in-person and online interviews and no other person was present, the interviewers’ gender may have shaped what women chose to share, particularly about family roles and daily routines.

Further analytic constraints should be noted. Because all interviews followed a semi-structured guide organised around five topic areas, the analysis cannot reliably separate content that participants raised spontaneously from content elicited by a direct prompt; the prominence of a topic in the dataset therefore reflects both participants’ concerns and the structure of the guide, which is reproduced in full in Supplementary File S1 so that readers can judge this for themselves. Several specific findings correspond to probes in the guide: the link between language and trust (Question 8), family help with setup (Question 5), smaller and gradual goals (Question 12), reminders around Asr and Maghrib (Question 14), optional sharing of progress with family (Question 15), and indoor alternatives in hot weather (Question 16). For these findings, the data show that participants endorsed and elaborated the issue when asked, not that they raised it unprompted. The four themes also correspond closely to the first four topic areas of the guide, which means the thematic structure partly reflects how the interviews were organised; the analytic contribution lies mainly in the organising concepts within each theme and in the patterns that cut across them (Section 3.6). Accounts relating to the fifth topic area, healthcare provider involvement, were not developed into a separate theme and appear only in summary form in Theme 1, which limits what the study can say about clinical integration (Section 4.6). In addition, although duration of program use varied from 4 to 16 weeks, a dataset of 14 accounts distributed across three duration bands is too small to establish whether perceptions differed systematically with length of exposure; any apparent patterning by duration would require a larger sample designed for that comparison. The same applies to diagnosis and prior digital experience: the sample included participants with different chronic conditions and levels of digital experience, but the numbers in each group are too small to establish whether perceptions differed systematically between them (Section 4.5).

Finally, the study reflects perceptions during a specific data-collection period (December 2025 to February 2026). Interviews took place in the cooler months, so accounts of summer heat drew on participants’ recollection and anticipation rather than on concurrent experience. As digital health programs and users’ digital literacy evolve, perceptions may also shift.

## 5. Conclusions

This qualitative descriptive study describes how adults with chronic diseases in Saudi Arabia who had used the physical activity function of the Sehhaty application for at least four weeks perceived it and formed expectations of digitally supported physical activity. These participants generally described the program as useful and acceptable while identifying barriers relating to usability, language, personalisation, cultural fit, and the psychological effects of numerical feedback. Perceived usefulness was conditional: the same feature could support one participant and discourage another, depending on health circumstances, digital confidence, family situation, and daily routine. Because all participants were sustained users recruited from tertiary centres in Riyadh, the findings describe acceptability among people who continued using a program; they do not establish population-level acceptability or effectiveness.

For developers and health services, the findings point to specific measures: fully Arabic interfaces with natural wording, reminders anchored to prayer times, heat-aware indoor alternatives, optional family sharing controlled by the patient, and goals that start from a person’s baseline and can be paused during illness.

Larger and more diverse samples across Saudi regions are needed, and future studies should deliberately recruit people who decline, discontinue, or struggle to use digital programs, since those perspectives are absent here. Longitudinal work is needed to examine how expectations and acceptability change over time and to test whether culturally adapted, clinically integrated design features improve engagement and patient-centred outcomes.

## Figures and Tables

**Table 1:** Participant demographic and clinical characteristics (*n* = 14).

Characteristic	Summary
Age, years: range	38–72
Age, years: mean (SD)	55.6 (11.1)
Gender: female/male	8/6
Diagnosis (categories not mutually exclusive)	
Type 2 diabetes mellitus	9 (64%)
Hypertension	8 (57%)
Cardiovascular disease	3 (21%)
Two or more concurrent conditions	5 (36%)
Disease duration, years: range	2–18
Disease duration, years: mean (SD)	9.0 (5.0)
Prior digital experience	
Minimal (calls and text messages only)	4 (29%)
Moderate (email, social media, basic applications)	6 (43%)
Extensive (multiple applications, frequent use)	4 (29%)
Duration of program use at interview	
4–8 weeks	5 (36%)
9–12 weeks	5 (36%)
13–16 weeks	4 (29%)
Nationality	
Saudi	11 (79%)
Expatriate resident	3 (21%)

SD, standard deviation. Percentages are of the total sample (*n* = 14) and are rounded to the nearest whole number, so subtotals may sum to 101%. Diagnosis categories are not mutually exclusive: participants with more than one chronic condition are counted in each applicable row, so the diagnosis rows sum to more than 100%.

**Table 2:** Thematic structure and participants quoted for each subtheme.

Theme	Subtheme	Participants Quoted
1. Reframing the digital presence: shifting expectations of self-care	Companionship and non-judgemental presence	P3, P7, P10, P11
	Gap between initial and actual functionality	P5; P9 (extract in Section 3.4)
2. The gap between perceived usefulness and everyday usability	Interface and navigation	P1, P12, P13
	Language and localisation	P8
3. Numbers as motivation and mirror: how feedback shapes self-perception	Motivational enhancement	P2, P10
	Contextual discouragement	P9
	Expectations of realistic, incremental goals	P14
4. Fitting digital physical activity programs into Islamic practice, family life, and the local environment	Prayer schedule integration	P4, P11
	Family-centred engagement	P6, P12
	Climate and environmental constraints	P7, P13

P, participant.

**Table 3:** Themes, participants’ accounts, and implications for the design and implementation of digital physical activity programs.

Theme	What Participants Reported	Design and Implementation Implication
1. Reframing the digital presence	Gentle, non-judgemental reminders and feedback felt like support between visits; some expected more tailored advice than the program could give	Keep reminders and feedback non-judgemental; state clearly at the clinic introduction what the program can and cannot do
2. Perceived usefulness versus everyday usability	Confusing navigation, crowded screens, English labels and awkward Arabic; reliance on family for setup	Simplify the main screen; provide a fully Arabic interface with wording reviewed by Saudi users; test with low-experience users and offer supported setup
3. Numbers as motivation and mirror	Graphs motivated some participants but discouraged others during illness; large fixed targets felt unrealistic	Set goals from the user’s recent baseline with small increments; allow illness pauses by the user or clinician; offer trend views as an alternative to daily totals
4. Islamic practice, family life, and the local environment	Reminders at unsuitable times; wish for family involvement alongside independence; reminders that did not account for extreme heat	Offer prayer-time reminder presets; provide optional, patient-controlled family sharing; switch to indoor activity suggestions when outdoor heat is high

Implications are design hypotheses derived from the accounts of sustained users; none was tested in this study.

## Data Availability

The interview transcripts are not publicly available due to participant consent restrictions and the potential for identification from contextual details. De-identified excerpts may be available from the corresponding author upon reasonable request, subject to Institutional Review Board approval.

## Supplementary Materials

The following supporting information can be downloaded: https://www.mdpi.com/article/10.3390/healthcare14193334/s1, Supplementary File S1, COREQ 32-Item Checklist and Interview Guide (including the complete 20-question semi-structured interview guide and probes).
