# Supplementary material for: Perceptions, Expectations, and Acceptability of Digital Physical Activity Programs Among Adults with Chronic Diseases in Saudi Arabia: A Qualitative Descriptive Study
Source: Healthcare (Basel). 2026 Oct 6;14(19):3334. doi: 10.3390/healthcare14193334 (PMC13665020; doi:10.3390/healthcare14193334)
Supplement: Supplementary file 1 [file healthcare-14-03334-s001.zip › healthcare-4587566-supplementary.pdf]

COREQ 32-Item Checklist and Interview Guide

Supplementary File S1

Part A. COREQ 32-Item Checklist

Item	COREQ criterion	Reported/support in final manuscript	Location
1	Interviewer/facilitator	J.M.A. and A.M.A. conducted the interviews.	Section 2.2 (Research Team and Reflexivity), paragraph 1; Section 2.5 (Data Collection), paragraph 1
2	Credentials	Both interviewers were PhD-qualified faculty members.	Section 2.2, paragraph 1
3	Occupation	Both were faculty members at King Saud University.	Section 2.2, paragraph 1
4	Gender	Both interviewers were male.	Section 2.2, paragraph 1
5	Experience and training	Five to ten years of qualitative research experience; formal training in qualitative interviewing and reflexive thematic analysis; prior experience interviewing adults with chronic disease.	Section 2.2, paragraph 1
6	Relationship established	Neither interviewer had a prior clinical, professional, or personal relationship with any participant.	Section 2.2, paragraph 1
7	Participant knowledge of interviewer	Participants were informed of the interviewers' institutional affiliation and research role and that the interviewers were not involved in clinical care.	Section 2.2, paragraph 1
8	Interviewer characteristics	Exercise-science and nursing backgrounds and initial assumptions about digital support were documented and examined through reflexive journals and team discussions.	Section 2.2, paragraph 2

9	Methodological orientation and theory	Qualitative descriptive design, critical realist stance, and Braun and Clarke's reflexive thematic analysis.	Section 2.1 (Study Design and Methodological Orientation), paragraphs 1–2
10	Sampling	Purposive sampling sought variation in age, gender, chronic disease type, and prior digital experience.	Section 2.3 (Setting, Sampling, and Recruitment), paragraph 1
11	Method of approach	Participants were recruited through clinic nursing staff and posters in waiting areas.	Section 2.3, paragraph 1
12	Sample size	Fourteen participants were interviewed.	Section 2.3, paragraph 3; Section 2.4 (Participants), paragraph 1; Figure 1
13	Non-participation	Eighteen individuals were approached; four declined during stage 2; reasons were not recorded. Eight of 26 screened individuals were excluded before approach.	Section 2.3, paragraph 3; Figure 1
14	Setting of data collection	Interviews were conducted in person at study sites or online by videoconference according to participant preference.	Section 2.5, paragraph 1
15	Presence of non-participants	Only the participant and interviewer were present.	Section 2.5, paragraph 1
16	Description of sample	Age, gender, diagnoses, disease duration, digital experience, program-use duration, and nationality are reported.	Section 2.4, paragraph 1; Table 1
17	Interview guide	A piloted semi-structured guide comprised 20 open-ended questions and probes across five topic areas; the complete guide appears in Part B below.	Section 2.5, paragraph 2; Supplementary File S1, Part B
18	Repeat interviews	Each participant was interviewed once; no repeat interviews were conducted.	Section 2.5, paragraph 1
19	Audio/visual recording	Interviews were audio-recorded with participant permission.	Section 2.5, paragraph 1; Section 2.9, paragraph 1

20	Field notes	Field notes were recorded during and immediately after interviews.	Section 2.5, paragraph 1
21	Duration	Interviews lasted 45–75 min.	Section 2.5, paragraph 3
22	Data saturation	Formal saturation criteria were not used; dataset adequacy was judged in relation to the research questions, consistent with reflexive thematic analysis.	Section 2.3, paragraph 4
23	Transcripts returned	Full transcripts were not returned to participants for comment or correction.	Section 2.8 (Rigour and Trustworthiness), Credibility paragraph
24	Number of data coders	Two analysts (J.M.A. and A.M.A.) each coded all 14 transcripts independently. The two code sets were compared at analytic meetings; differing readings of the same extract were recorded in memos and tested against the full dataset, and the final structure was agreed between the analysts and discussed with the wider team. Coding agreement statistics were not used.	Section 2.7 (Data Analysis), paragraph 1, Phases 2 and 3, and the paragraph preceding Figure 2
25	Description of coding tree	Ten candidate categories were refined into four final themes; the codebook, thematic matrix, and worked examples are available from the corresponding author on reasonable request.	Section 2.7, Phase 6 and the two paragraphs preceding Figure 2; Figure 2; Data Availability Statement
26	Derivation of themes	Coding was primarily inductive; themes were actively developed through iterative reflexive thematic analysis rather than predetermined.	Section 2.7, Phase 2
27	Software	Data were managed manually using Microsoft Word and Microsoft Excel.	Section 2.7, data-management paragraph

28	Participant checking	Preliminary themes and illustrative quotations were shared with four participants who had agreed to re-contact; feedback was treated as an additional interpretive perspective.	Section 2.8, Credibility paragraph
29	Quotations presented	Illustrative quotations are presented and attributed using coded participant identifiers (Participant 1–Participant 14 in the text; P1–P14 in Table 2).	Sections 3.2–3.5; Table 2
30	Data and findings consistent	Results are organised into four themes and subthemes, with quotations and summaries aligned to the thematic structure.	Sections 3.1–3.6; Table 2; Figure 2
31	Clarity of major themes	Four major themes are explicitly named, described, and linked to the research questions.	Sections 3.1–3.5; Table 2
32	Clarity of minor themes/diverse cases	Subthemes and divergent/disconfirming cases are reported, including variation in numerical feedback, family involvement, and cultural adaptation.	Sections 3.2–3.6

Part B. Semi-Structured Interview Guide

Thank the participant, confirm informed consent and permission to audio-record, remind the participant that participation is voluntary and that any question may be skipped, and explain that the discussion concerns their experiences, perceptions, expectations, and acceptability of the digital physical activity program.

Topic Area 1. Initial perceptions and expectations on learning about the program

1. Can you tell me what you first thought when you learned that you would be using this digital physical activity program?

   Probe: What did you expect it to do for you?

   Probe: Did anything concern or interest you at the beginning?

2. Before you started using it, what did you think the main purpose of the program would be?

   Probe: Did you see it mainly as a step counter, a source of advice, a connection with healthcare providers, or something else?

3. What benefits did you expect the program to provide for your health or physical activity?

   Probe: Were there any specific changes you hoped it would help you make?

4. How did your expectations before using the program compare with what you actually experienced?

   Probe: What was better than expected?

   Probe: What was less than expected or missing?

Topic Area 2. Application navigation, interface design, and usability

5. Please describe what it was like to learn how to use the application.

   Probe: Which parts were easy or difficult to learn?

   Probe: Did anyone help you?

6. How easy or difficult is it for you to find the information or features you need?

   Probe: Are there screens, buttons, menus, or steps that are confusing?

7. What do you think about the amount and arrangement of information shown on the screen?

   Probe: Is there too much, too little, or about the right amount?

   Probe: What would you simplify or change?

8. How well does the language used in the application work for you?

   Probe: Are the Arabic wording and translations natural and understandable?

   Probe: Are any features or terms still in English?

Probe: Does the language affect your trust in the program?

Topic Area 3. Activity tracking and feedback mechanisms

9. How do you feel about the program tracking and showing your daily steps or activity?

Probe: Does seeing the number change what you do?

Probe: How often do you check it?

10. What is your experience of the graphs, progress displays, or achievement notifications?

Probe: Do they encourage you, discourage you, or make little difference?

Probe: Can you give an example?

11. How do you feel when the program shows that you have not met an activity goal?

Probe: Does your reaction change when illness, pain, fatigue, or another circumstance affects your activity?

12. What kind of goals and feedback would feel realistic and helpful to you?

Probe: Would smaller or gradual goals be more useful?

Probe: How should the program respond when your health temporarily limits activity?

Topic Area 4. Integration into daily routines and cultural, religious, familial, and environmental contexts

13. How does the program fit into your normal daily routine?

Probe: When do you usually look at it or act on its reminders?

Probe: Are there times when it does not fit well?

14. How, if at all, should reminders or activity suggestions take prayer times into account?

Probe: Would you prefer reminders before or after particular prayers such as Asr or Maghrib?

Probe: Should you be able to set this easily?

15. What role, if any, does your family play in your use of the program?

Probe: Do family members help with setup or use?

Probe: Do they encourage you or discuss your progress?

Probe: Would you want an optional way to share progress with them?

16. How do weather and the local environment affect your ability to follow the program’s activity suggestions?

Probe: What happens during very hot weather?

Probe: What indoor or alternative activities would be useful?

Topic Area 5. Healthcare provider involvement, perceived benefits, acceptability, and improvements

17. What role do you expect healthcare professionals to have in a digital physical activity program?

    Probe: Would you want them to review your progress, answer messages, adjust goals, or provide advice?

    Probe: How quickly would you expect a response?

18. Overall, what has been most useful or beneficial about using the program?

    Probe: Has it changed how you think about physical activity or self-care?

    Probe: Has it affected what you do between clinic visits?

19. What makes a digital physical activity program acceptable or unacceptable for you to continue using?

    Probe: Think about reliability, ease of use, language, privacy, cultural fit, family involvement, feedback, and health circumstances.

20. If you could redesign this program for adults with chronic diseases in Saudi Arabia, what would you change or add?

    Probe: What should definitely be kept?

    Probe: What should be removed or simplified?

    Probe: What would make you recommend it to someone else?

Closing prompt: Is there anything else about your experience, expectations, or views of digital physical activity programs that you think is important and that we have not discussed?
